# Cue overlap supports preretrieval selection in episodic memory: ERP evidence

**DOI:** 10.3758/s13415-021-00971-0

**Published:** 2021-12-29

**Authors:** Arianna Moccia, Alexa M. Morcom

**Affiliations:** School of Psychology, University of Sussex, Pevensey I, Falmer, BN1 9QH UK

**Keywords:** Proactive control, Event-related potentials, Episodic Memory, Recollection, Retrieval Orientation

## Abstract

People often want to recall events of a particular kind, but this selective remembering is not always possible. We contrasted two candidate mechanisms: the overlap between retrieval cues and stored memory traces, and the ease of recollection. In two preregistered experiments (*N*s = 28), we used event-related potentials (ERPs) to quantify selection occurring before retrieval and the goal states — retrieval orientations — thought to achieve this selection. Participants viewed object pictures or heard object names, and one of these sources was designated as targets in each memory test. We manipulated cue overlap by probing memory with visual names (Experiment 1) or line drawings (Experiment 2). Results revealed that regardless of which source was targeted, the left parietal ERP effect indexing recollection was selective when test cues overlapped more with the targeted than non-targeted information, despite consistently better memory for pictures. ERPs for unstudied items also were more positive-going when cue overlap was high, suggesting that engagement of retrieval orientations reflected availability of external cues matching the targeted source. The data support the view that selection can act before recollection if there is sufficient overlap between retrieval cues and targeted versus competing memory traces.

**Supplementary Information:**

The online version contains supplementary material available at 10.3758/s13415-021-00971-0.

## Introduction

We often want to retrieve a particular kind of event from memory. For example, we could check the reliability of a piece of information by recalling whether we heard it in conversation with friends or saw it on a news website. Ideally, we can selectively pull up relevant memories of news, without also recalling conversations with friends. To do this, selection needs to act before recollection occurs. Research suggests that people can only sometimes selectively remember in this way (Rosburg & Mecklinger, 2017). This ability tracks individual working memory capacity (Elward & Wilding, 2010), which in turn correlates with abilities on a range of other tasks reduced in later life (Dywan et al., 1998; Keating et al., 2017; Unsworth, 2016). Time-resolved measures of brain activity like electroencephalographic event-related potentials (ERPs) allow us to quantify proactive control processes that act prior to the point of retrieval and distinguish them from their impact on selective recollection. We investigated two factors proposed to be critical: the ease of retrieving targeted events (Herron & Rugg, 2003a), and the degree to which external memory cues overlap stored information (Hornberger et al., 2004).

Theories of memory assume that external cues and internal cognitive control are both important determinants of what can be brought to mind (Anderson & Bjork, 1994; Johnson & Raye, 1981; Tulving, 1983). According to the encoding specificity principle, effective retrieval cues are ones that reinstate part of the information stored in the memory trace (Tulving & Thomson, 1973; see also Morris, Bransford, & Franks, 1977). At the neural level, these partial cues are thought to initiate pattern completion by the hippocampus, which in turn triggers the cortical signals supporting recollection (Norman, 2010). The greater the *cue overlap *— similarity between the neural representations of the cue and the stored information — the more likely the information is to be retrieved (but see Nairne, 2002). For example, it is well established that reinstating the original context in which information was studied, such as a location, benefits memory (see Smith & Vela, 2001 for review), particularly when task demands require retrieval of context (Bramão & Johansson, 2018).

Internal control processes enabling selective retrieval have been theorized within several frameworks. For memory to guide behavior, people must not only remember information but also infer its source. This source monitoring involves controlled processes that allow us to weight, evaluate, and edit recovered information according to retrieval goals (Johnson & Raye, 1981). Importantly, some of these processes act before retrieval and are assumed to help select what information will be recovered (Burgess & Shallice, 1996; Johnson & Raye, 2000; see also Norman & Bobrow, 1979; Williams & Hollan, 1981; Tulving 1983). For example, in the context maintenance and retrieval model, memory search during free recall involves reinstatement of temporal or semantic context (Polyn et al., 2009). Jacoby and others also have proposed that internal pre-retrieval control constitutes a form of proactive “early selection” that may be more effective at preventing memory errors than reactive “late correction” processes, such as retrieval monitoring (Halamish et al., 2012; Jacoby, Kelley & McElree, 1999; Jacoby et al., 2005; Morcom, 2015). Indirect behavioral evidence for mental reinstatement comes from the finding that instructions enabling internal control can have similar effects to external cues: reinstating environmental context no longer boosts memory when participants can imagine the original context for themselves, suggesting that people can mentally reinstate context to trigger recall of desired information (Sahakyan & Kelley, 2002; Smith, 1979; Smith & Vela, 2001; Starns & Hicks, 2013). Jacoby et al. (2005) also developed a 3-stage behavioral procedure to measure mental reinstatement of “deeply” and “shallowly” encoded targeted sources without involving external cues. However, behavioral measures of pre-retrieval control are necessarily indirect, because mnemonic decisions are a function of multiple processes that occur before, during, and after the point of retrieval. Measures of brain activity provide a powerful way to study these different stages of retrieval separately and shed light on the underlying neural operations (Mecklinger, 2010; Polyn, 2005; Rugg & Wilding, 2000).

We used ERPs to quantify the retrieval of incidental and targeted information, as well as the goal states on which selection depends. In the recognition exclusion task (Jacoby, 1991), people study items in two sources, such as picture and word formats. They are then asked to focus retrieval on just one source at a time, accepting as targets only items from that source (e.g., those studied as pictures), and rejecting both items from the other source (non-targets, e.g. those studied as words) and unstudied (new) items. To do the task efficiently, participants need only to recollect items from the targeted source. Selective recollection is measured by comparing the left parietal ERP old/new effect for targets and non-targets (Dywan et al., 1998). This positive-going ERP modulation approximately 500-800 ms after the retrieval cue is well established as an index of recollection as opposed to familiarity in recognition tests, being larger when source memory is successful than unsuccessful, as well as when participants report subjective recollection rather than familiarity (Duzel et al., 1997; Rugg & Curran, 2007; Wilding et al., 1995). It also is larger when more information is recollected (Leynes & Mok, 2017) or recollected source information has greater precision (Vilberg & Rugg, 2009). When recollection is selective, the left parietal effect is larger for targets than non-targets, and non-target activity may be indistinguishable from new (for meta-analysis see Rosburg & Mecklinger, 2017). This demonstrates that selection has occurred before retrieval, converging with evidence from retrieval inhibition tasks showing that the left parietal effect is reduced when retrieval is prevented (Bergström et al., 2007).

Brain imaging also can be used to probe the goal-directed processes assumed to bring about selective remembering. The cognitive operations that bias memory search in the service of goals are referred to as “retrieval orientations” (Rugg & Wilding, 2000). By adopting a retrieval orientation, people modify how external retrieval cues are processed depending on the targeted source, for example by reinstating encoded context as outlined above. We distinguished these goal states from successful retrieval by comparing ERPs for correctly rejected new items under different retrieval goals (Rugg & Wilding, 2000). Studies using this approach have suggested that people can orient retrieval to a range of sources (Hornberger et al., 2006; Johnson & Rugg, 2006; Morcom & Rugg, 2012; Ranganath et al., 2000; Ranganath & Paller, 1999; Robb & Rugg, 2002). A few of these studies have also analyzed neural correlates of successful retrieval, finding significant retrieval orientation effects and target-selective recollection in one task condition and non-significant retrieval orientation effects and non-selective recollection in another (Dzulkifli et al., 2006; Dzulkifli & Wilding, 2005; and with fMRI data, McDuff et al., 2009; but see Herron & Rugg, 2003a and Rosburg et al., 2013, 2014 for significant retrieval orientation effects despite non-selective recollection in one condition). Such findings support the assumption that retrieval goal states are important for goal-relevant recall, but do not reveal what factors enable these processes to operate.

According to one view, selective recollection is only possible when target retrieval is easy (Herron & Rugg, 2003a). Several ERP studies using the exclusion task have demonstrated that non-target left parietal effects were more prominent when target accuracy was reduced, for example, by manipulating study-test delay (Dzulkifli et al., 2006; Herron & Wilding, 2005), study list length (Wilding et al., 2005), or the encoding task (Herron & Rugg, 2003b; Rosburg et al., 2011, 2013, 2014). These studies suggest that the ease of retrieving targets may influence whether pre-retrieval selection occurs (Evans et al., 2010; Herron & Wilding, 2005; Rosburg, Mecklinger, & Johansson, 2011; Rosburg et al., 2013, 2014; Wilding et al., 2005). Relative target accuracy compared to non-targets also may be important; when non-targets are easier to recall, target recollection is typically not prioritized (Rosburg et al., 2011). However, accuracy does not seem to be the whole story. Larger left parietal effects to targets than non-targets, with no significant difference between non-targets and new, are sometimes found even when target accuracy is low (Evans et al., 2010; Herron & Wilding, 2005; Sprondel, Kipp & Mecklinger, 2012).

An alternative proposal is that the ability to recollect selectively depends on how memory is cued and is made possible by cues that overlap with the targeted source. In an ERP study of retrieval orientation, Hornberger et al. (2004) found more positive-going retrieval orientation effects when people used visual word cues to retrieve visual words compared with pictures, and when they used picture cues to retrieve pictures compared to visual words. Thus, the engagement of goal-related brain activity tracked the overlap between test cues and studied items (see also Bramão et al., 2017). These researchers did not investigate the ERP correlates of retrieval success in that study, but there is preliminary evidence suggesting that cue overlap also has downstream consequences for recollection. Two studies found a target-selective left parietal ERP effect only when memory was probed with cues that exactly matched the target format, e.g., test cues and targets were visual words while non-targets were pictures (Herron & Rugg 2003a; Stenberg, Johansson, & Rosén 2006; see also Morcom & Rugg, 2012). However, these studies also varied the encoding tasks that participants performed when studying the pictures and words, so it is unknown whether participants could selectively remember based on only the studied format (but see Stenberg et al., 2006). Most importantly, we do not know whether the degree of overlap between external cues and targets remains critical for selection when cues and targets are not identical, i.e., when they are not “copy-cues.”

The main goal of the present studies was to investigate whether cue-target overlap would enable selective recollection even when overlap is incomplete. Although this proposal and the ease of target recollection account are not mutually exclusive, because cue-target overlap is a factor in ease of recollection, we were able to tease them apart experimentally by testing whether recollection is selective when performance was consistently better for targets from one source, regardless of which was targeted. We also wanted to determine whether cue overlap effects on selectivity generalized to different retrieval cues. In two preregistered ERP experiments, participants had to remember either words they had heard or pictures they had seen, in separate blocks. In Experiment 1, test cues were visual words, which overlapped more with the auditory word source. Supporting the overlap view, the left parietal effect was larger for targets than non-targets only when auditory words were targeted. In Experiment 2, we used object line drawings as cues, which overlapped more with the picture source (Czernochowski et al., 2005). The findings confirmed a complementary asymmetry to Experiment 1, with greater selectivity again for the high-overlap source. In both experiments, performance was better for the picture source, so the results cannot be explained by the ease of target recollection. As expected, the direction of ERP retrieval orientation effects also reflected the degree of cue-target overlap. Together these findings show that cue overlap enables selection prior to recollection, as predicted by the encoding specificity principle.

## Methods

### Participants

Twenty-eight participants were included in Experiment 1 (20 females, age *M* = 22.79 years, *SD* = 4.14) and another 28 in Experiment 2 (20 females, age *M* = 24.57 years, *SD* = 3.71). One further participant in Experiment 2 was excluded due to an insufficient number of artefact-free trials (for preregistered criteria see https://osf.io/j84z6 and https://osf.io/pqn4z). Sample sizes were determined *a priori* using effect sizes from Dzulkifli and Wilding (2005). Power analysis using G*Power 3.1.9.2 indicated that 29 and 17 participants would be required respectively to replicate the smallest main effect of retrieval orientation, from 500-600 ms (*d* = 1.4) and the main effect of target versus non-target left parietal effects from 500-800 ms (*d* = 1.9) with .95 power at *α* = .05. *N* was rounded to 28 to simplify counterbalancing. Participants were recruited from the University of Edinburgh student population. They were right-handed with normal or corrected-to-normal vision and hearing, who were in good self-reported health and not taking medication that might affect cognition. All were very fluent in English (self-rated scores ≥15/20 on ratings adapted from Vega-Mendoza et al. (2015) (see https://osf.io/gcrm2). The experiments were approved by the Psychology Research Ethics Committee at the University of Edinburgh, ref.: 135-1819/1 and 300-1819/1. Participants were compensated either with university credits or money for their participation.

### Materials

**Fig. 1:**
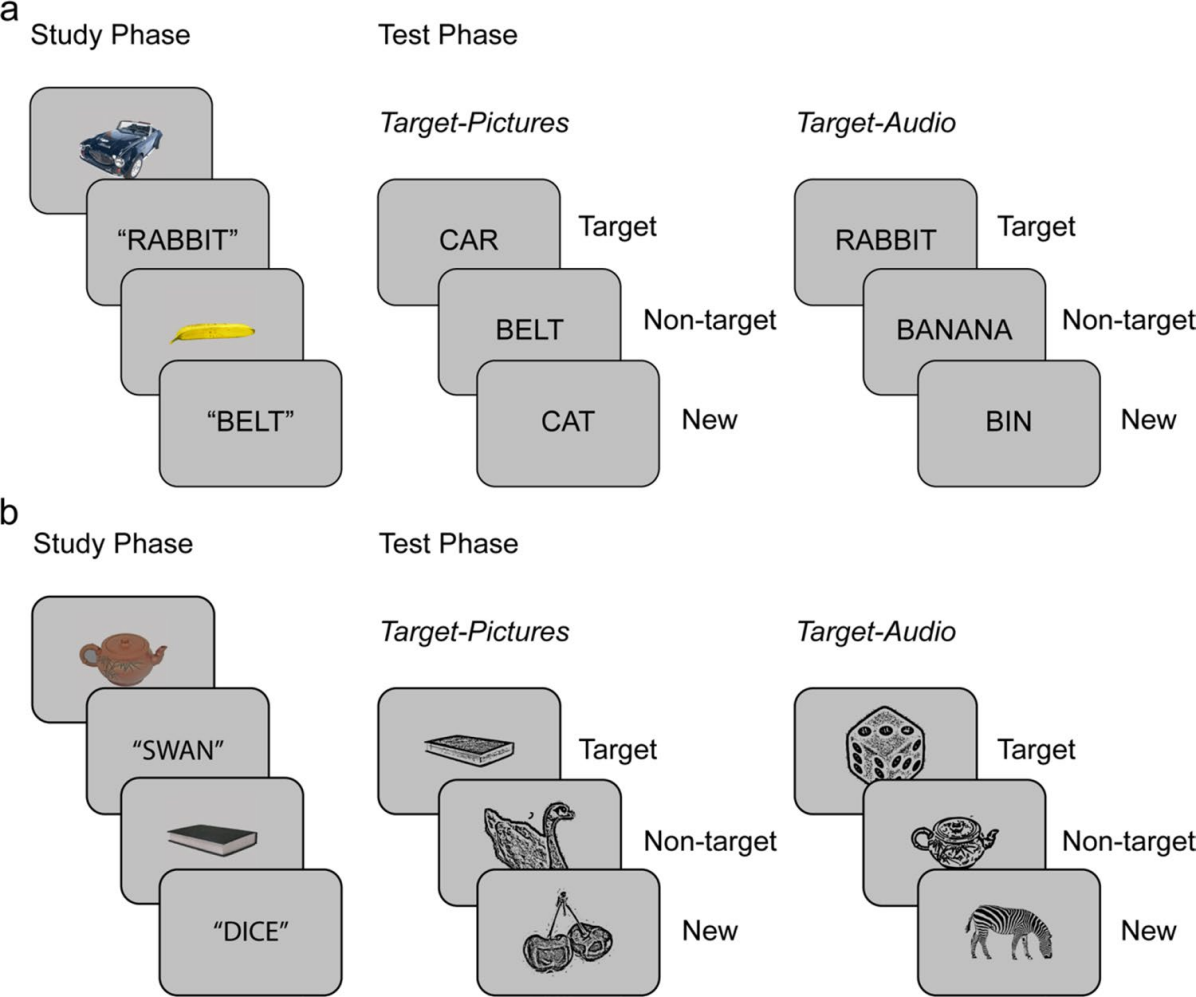
Experimental paradigm: recognition exclusion task procedure for Experiments 1 (a; visual word test cues) and Experiment 2 (b; line drawing test cues). In both experiments, participants studied a single block of pictures and auditory words. In each of the two test blocks, either studied pictures or studied auditory words were targets. Required responses were “yes” to the target items and “no” to non-target and new items (see Materials and Procedure for details)

Stimuli in both experiments were pictures and names of 240 common objects (Figure 1). Study phase stimuli appeared as either colored pictures or as auditory words spoken by an English native male voice. At test, memory probes were visual words in Experiment 1 or grey-scale line drawings in Experiment 2. The audio files were a subset of those used by Hornberger et al. (2004). Corresponding object images were sourced from the BOSS database (Brodeur et al., 2014), POPORO database (Kovalenko et al., 2012), or online (see Supplemental Material available online). The critical items were divided into six sets of 40 items each. For each of the two study-test cycles, one set of pictures and one of auditory words were combined to create a study list of 80 items. The corresponding visual words (Experiment 1) or line drawings (Experiment 2) were then combined with a third set of new items to create the test list of 120 items. For each study-test cycle, half of the studied pictures, half of the studied auditory words, and half of the new items were allocated to the first test block and the remainder to the second test block. In total, there were 80 critical targets, 80 critical non-targets, and 80 critical new items. Two further filler pictures were added at the beginning of each study list and two unstudied filler items at the beginning of each test block. An additional 12 items served in practice lists. Item presentation order was determined randomly within each study and test list.

### Procedure

Each experiment consisted of two study-test cycles (Fig. 1), during which the EEG was recorded.

#### Study phase

Participants studied items presented as pictures or auditory words. Pictures appeared at the center of a square frame on a grey background subtending a visual angle of 4.32°. Auditory words were played at 44,100 Hz, while a blank screen was shown. On each trial, a preparatory cue signaled the format of the upcoming item, either a yellow asterisk “*” or a blue lowercase “o” (allocation to pictures and auditory words was counterbalanced). Participants were instructed to learn the items for a subsequent memory test, while judging their pleasantness: “very pleasant,” “somewhat pleasant,” “pleasant,” or “not pleasant.” To maximize differences in processing between stimulus formats, they were instructed to take into account the holistic experience, paying attention to the visual or acoustic features. The preparatory cues were on-screen for 1,000 ms, followed by a blank screen for 100 ms, then stimulus presentation for 1,000 ms. A red fixation cross followed for 1,500 ms before the word “RESPOND” was presented at the center for up to 3,000 ms, during which participants were asked to respond by pressing one of the keys on the keyboard. A 100-ms blank screen separated participants’ response and the next trial.

#### Test phase

Each test phase comprised two blocks with different target designations. In each, items presented in one format at encoding were designated as targets (Target-Pictures or Target-Audio). For example, in the Target-Pictures block, participants were instructed to answer “yes” to an item if they had seen a picture of a corresponding object in the preceding study phase and “no” to all other items. Target designation switched for the second test block and also was signaled on each trial using the same preparatory symbols as at study. All items appeared in the middle of the computer screen. In Experiment 1, test probes were visual words shown in 48-pt, black uppercase letters. In Experiment 2, test probes were grey-scale line drawings, presented with a 3.71° visual angle. Test trials began with pre-cues for 500 ms, followed with a black fixation for 1,800 ms before stimulus presentation. Stimuli appeared for 3,000 ms followed by a red fixation for 500 ms in Experiment 1, and for 500 ms followed by a 3,000-ms fixation in Experiment 2. Participants’ responses were recorded during stimulus presentation in Experiment 1 and during fixation in Experiment 2. Participants were instructed to fixate in the middle of the screen throughout stimulus presentation, even after a response was made, to avoid excessive ocular movements.

The order of Target-Picture and Target-Audio blocks was counterbalanced across participants. Keypress responses used middle and index fingers at study, and index fingers at test, and the allocation of judgments to left and right hands was counterbalanced. The main experiment was preceded by a short practice phase, and study and test phases were separated by a 1- to 5-min interval, during which participants completed a distractor task consisting of 12 pen-and-paper true or false questions.

### EEG recording and pre-processing

EEG data were recorded with a BioSemi Active Two AD-box with 24-bit signal digitization from 64 active silver/silver chloride electrodes embedded in an elastic cap using the extended International 10-20 system configuration (Nuwer et al., 1998; http://www.biosemi.com/products.htm). Common Mode Sense and Driven Right Leg electrodes worked as ground electrode and noise rejection conjointly, while bipolar electrodes, placed above and below the right eye and on the outer canthi, recorded vertical and horizontal eye movements (electrooculogram; EOG). EEG and EOG signals were acquired continuously at a 1,024-Hz sampling rate with amplifier bandwidth of 0 ± 208 Hz (3 dB) and referenced to the CMS reference electrode. EEG data were preprocessed using the EEGLAB toolbox (Delorme & Makeig, 2004) in MATLAB R2018a. Data were first re-referenced offline to the average of the left and right mastoid electrodes. A 0.1-40 Hz Hamming windowed-sinc FIR filter was applied with a 50-Hz notch filter for line noise. Data were divided into 4,500-ms study and 6,700-ms test epochs, time-locked to the stimulus onset. Customized threshold functions from the FASTER toolbox (Nolan et al., 2010) were used to identify and reject epochs and channels with excessive gross artefacts. The preprocessing pipeline and threshold criteria were developed for Experiment 1 and pre-registered for Experiment 2. Criteria were based on participant-level *z*-transformed values over trials that exceeded ±3 (see Supplemental EEG preprocessing online for details). Independent Component Analysis (ICA) was used to correct for EOG artefacts, by manually removing ICA components attributable to vertical and horizontal eye movements. Rejected channels were then replaced by interpolation using data from neighboring electrodes. A 200-ms pre-stimulus baseline was used for ERP computation.

### Statistical analysis

All reported behavioral and ERP analyses were pre-registered. Statistical analyses were conducted in R 3.6.1 (R Core Team, 2019) except where stated and alpha was set at .05. In analyses of variance (ANOVAs), we applied a Greenhouse-Geisser nonsphericity correction where appropriate. Benjamini-Hochberg false discovery rate (FDR) multiple comparison corrections (Benjamini & Hochberg, 1995) were used in *post hoc* tests following significant interactions, and all reported *p* values are adjusted. Cohen’s *d* was calculated by dividing mean differences by the pooled standard deviation (Dunlap et al., 1996).

## Results

### Exclusion task performance

We assessed differences in performance according to target designation for targets (items studied as pictures in the Target-Pictures condition or as auditory words in the Target-Audio condition), non-targets and new items (Table 1). We also examined participants’ ability to discriminate between picture and audio sources using discrimination (*d*’) and criterion (C) measures between targets and non-targets (Snodgrass & Corwin, 1988). Source *d*’ was computed by subtracting the *z*-nontarget false-alarm score from the *z*-target hit score for the Target-Picture and Target-Audio conditions, and response bias (C) was calculated according to Macmillan and Creelman (1991). Before computing these sensitivity measures, raw trial numbers were corrected for a potential outcome of zero by adding 1 to the sum of old and the sum of new items and 0.5 to the target hits, non-target correct rejections (CRs), or non-target false alarms (Hautus, 1995; Snodgrass & Corwin, 1988).

**Table 1:** Recognition exclusion task performance

	Experiment 1			Experiment 2		
Target designation	Target	Non-target	New	Target	Non-target	New
Target-audio						
Accuracy	.78	.90	.87	.71	.94	.81
	[.74, .81]	[.88, .93]	[.84, .91]	[.67, .76]	[.92, .96]	[.76, .86]
RTs	1200	1257	1264	1315	1188	1529
	[1139, 1261]	[1222, 1293]	[1199, 1,330]	[1263, 1367]	[1136, 1240]	[1467, 1592]
Source *d*’	2.19			2.19		
	[1.99, 2.39]			[2.02, 2.36]		
Response bias (C)	0.29			0.50		
	[0.20, 0.38]			[0.42, 0.58]		
Target-Picture						
Accuracy	.81	.86	.92	.89	.88	.91
	[.78, .85]	[.83, 0.90]	[.89, .94]	[.86, .91]	[.85, .91]	[.89, .93]
RTs	1097	1308	1294	1062	1297	1310
	[1028, 1165]	[1249, 1367]	[1224, 1363]	[1008, 1116]	[1259, 1335]	[1234, 1385]
Source *d*’	2.14			2.45		
	[1.94, 2.34]			[2.28, 2.62]		
Response bias (C)	0.13			−0.01		
	[0.04, 0.22]			[−0.09, 0.07]		

Note: Table shows means and [95% confidence intervals] of accuracy proportions and median response times (RTs in ms) for correctly identified items according to trial type, target designation and experiment. Confidence intervals were adjusted using the Cousineau-Morey method for within-subject variables (Morey, 2008).

#### Accuracy

In Experiment 1, when retrieval cues were words, responses were generally more accurate for studied pictures, whether these were identified as targets (Target-Pictures) or non-targets (Target-Audio). ANOVA on accuracy proportions with factors of Item Type (targets/non-targets/new) and Target Designation (picture/audio) revealed a significant main effect of Item Type, *F*(1.59, 42.83) = 17.66, *p* < .001, *η*^*2*^_*p*_ = .395, a non-significant main effect of Target Designation, *F*(1, 27) = 0.90, *p* = .352, *η*^*2*^_*p*_ = .032, and a significant interaction, *F*(1.86, 50.34) = 5.35, *p* = .008, *η*^*2*^_*p*_ = .165. Although pairwise *post hoc t*-tests did not show significant effects of target designation in any condition, there was a slight accuracy advantage for items studied as pictures both for targets, *t*(27) = 1.91, *p* = .067, *d* = 0.40 and non-targets, *t*(27) = −1.84, *p* = .076, *d* = 0.49, and for new items when targeting the pictures, *t*(27) = 1.79, *p* = .084, *d* = 0.48. We also checked directly whether source discrimination differed according to target designation. Paired-sample *t*-tests showed no significant difference in *d’*, *t*(27) = 0.38, *p* = .705, *d* = 0.10, but response criterion was significantly more conservative, *t*(27) = 2.65, *p* = .013, *d* = 0.71. Thus, participants were more likely to give “no” responses when identifying auditory than picture targets.

In Experiment 2, when retrieval cues were line drawings, accuracy was again greater for items studied as pictures. ANOVA with the same factors revealed significant main effects of Item Type, *F*(1.66, 44.78) = 14.67, *p* < .001, *η*^*2*^_*p*_ = .352 and Target Designation, *F*(1, 27) = 33.95, *p* < .001, *η*^*2*^_*p*_ = .557, and a significant interaction *F*(1.81, 48.89) = 30.31, *p* < .001, *η*^*2*^_*p*_ = .529. *Post hoc t*-tests confirmed more accurate identification of both targets and non-targets when they were studied as pictures than as auditory words, *t*(27) = 7.86, *p* < .001, *d* = 1.71, and *t*(27) = −3.75, *p* = .001, *d* = 0.88. New items were better identified when participants were targeting pictures, *t*(27) = 3.93, *p* = .001, *d* = 0.97. The analysis of source discrimination using *d’* also revealed a memory advantage for pictures as targets. Participants were significantly better at discriminating source when targeting pictures than auditory words, *t*(27) = 2.36, *p* = .026, *d* = 0.63. As in Experiment 1, response criterion was significantly more conservative when remembering the auditory targets, *t*(27) = 9.23, *p* < .001, *d* = 2.47.

#### Response times

We expected that participants would respond more slowly to non-targets than targets irrespective of targeted format — a pattern thought to suggest prioritization of target retrieval (Rosburg and Mecklinger, 2017). We analyzed median RTs for trials attracting correct responses. When cues were words in Experiment 1, ANOVA with factors of Item Type (target hits/nontarget CRs/new CRs) and Target Designation (picture/audio) revealed a significant main effect of Item Type, *F*(1.47, 39.82) = 10.06, *p* = 0.001, *η*^*2*^_*p*_ = 0.272, a non-significant main effect of Target Designation, *F*(1, 27) = 0.08, *p* = 0.774, *η*^*2*^_*p*_ = 0.003, and a significant interaction, *F*(1.55, 41.97) = 7.41, *p* = 0.001, *η*^*2*^_*p*_ = 0.215. Post hoc *t*-tests confirmed that target responses were faster when pictures as opposed to auditory words were correctly identified, *t*(27) = −2.6, *p* = 0.015, *d* = 0.61, whereas RTs for non-targets and new items did not differ significantly by target designation, *t*(27) = 1.45, *p* = 0.158, *d* = 0.41, and *t*(27) = 0.79, *p* = 0.434, *d* = 0.18.

Responses also were faster for items studied as pictures than auditory words in Experiment 2. ANOVA with the same factors revealed significant main effects of Item Type *F*(1.32, 35.72) = 28.80, *p* < 0.001, *η*^*2*^_*p*_ = 0.516, and Target Designation *F*(1, 27) = 27.07, *p* < 0.001, *η*^*2*^_*p*_ = 0.501, as well as a significant interaction *F*(1.92, 51.79) = 40.40, *p* < .001, *η*^*2*^_*p*_ = 0.599. *Post hoc t-*tests showed that participants were significantly faster when identifying items studied as pictures than auditory words, whether these were targets or non-targets, *t*(27) = − 9.03, *p* < 0.001, *d* = 1.85 and *t*(27) = 3.21, *p* = .003, *d* = 0.93. In parallel with the findings for accuracy, responses to new items were also significantly faster when targets were pictures vs. auditory words, *t*(27) = −5.39, *p* < 0.001, *d* = 1.60. Thus, not only were participants better (more accurate) at identifying studied pictures than auditory words, but they also identified the picture items faster, despite the higher cue-target overlap with the auditory source in Experiment 1.

### ERP results

To test our principal hypotheses about the selectivity of target over non-target recollection, we examined the left parietal old/new effect in a focal analysis restricted to data from three parietal electrodes (P1/P3/P5) from 500 to 800-ms post-stimulus, following Dzulkifli and Wilding (2005). We quantified the mean per-participant stimulus-locked ERP amplitudes for correct responses in each experimental condition (target hits, non-target CRs, and new CRs) according to target designation (Target-Pictures and Target-Audio). These analyses were complemented by subsidiary, global analyses, which tested whether ERPs evoked by targets and non-targets differed outside the predefined locations and time-windows. The global analyses included all electrodes and timepoints from 300 to 1,400-ms post stimulus, with a family-wise error correction using the nonparametric cluster permutation method from the FieldTrip toolbox (Maris & Oostenveld, 2007; Oostenveld et al., 2011). Thus, we i) ran dependent *t*-tests on the contrast of interest at each electrode and time-point; ii) defined clusters of temporally and spatially adjacent samples significant at *α* = .05 (sample-level *α*); iii) computed a cluster-level statistic equal to the sum of *t*-values per cluster; and iv) evaluated the maximum difference of this cluster-level statistic under its permutation distribution, created by randomly swapping data points between conditions within participants. We used 5,000 randomization draws to estimate each *p*-value (1-tailed cluster-level *α* of .025).

To assess retrieval orientation effects reflecting retrieval goal-states, we compared ERPs elicited by new CRs according to target designation. The focal analyses used a 3 x 3 grid of electrode locations (F5, Fz, F6/C5, Cz, C6/P5, Pz, P6) in three epochs: 300-600 ms, 600-900 ms, and 900-1,200 ms, following Hornberger et al. (2004). Global analyses also were conducted following the above procedure. Additional analyses of retrieval orientation effects time-locked to the preparatory cues did not yield significant results and are included in *Supplemental Results* available online.

### Recollection selectivity

#### Focal analyses

**Fig. 2:**
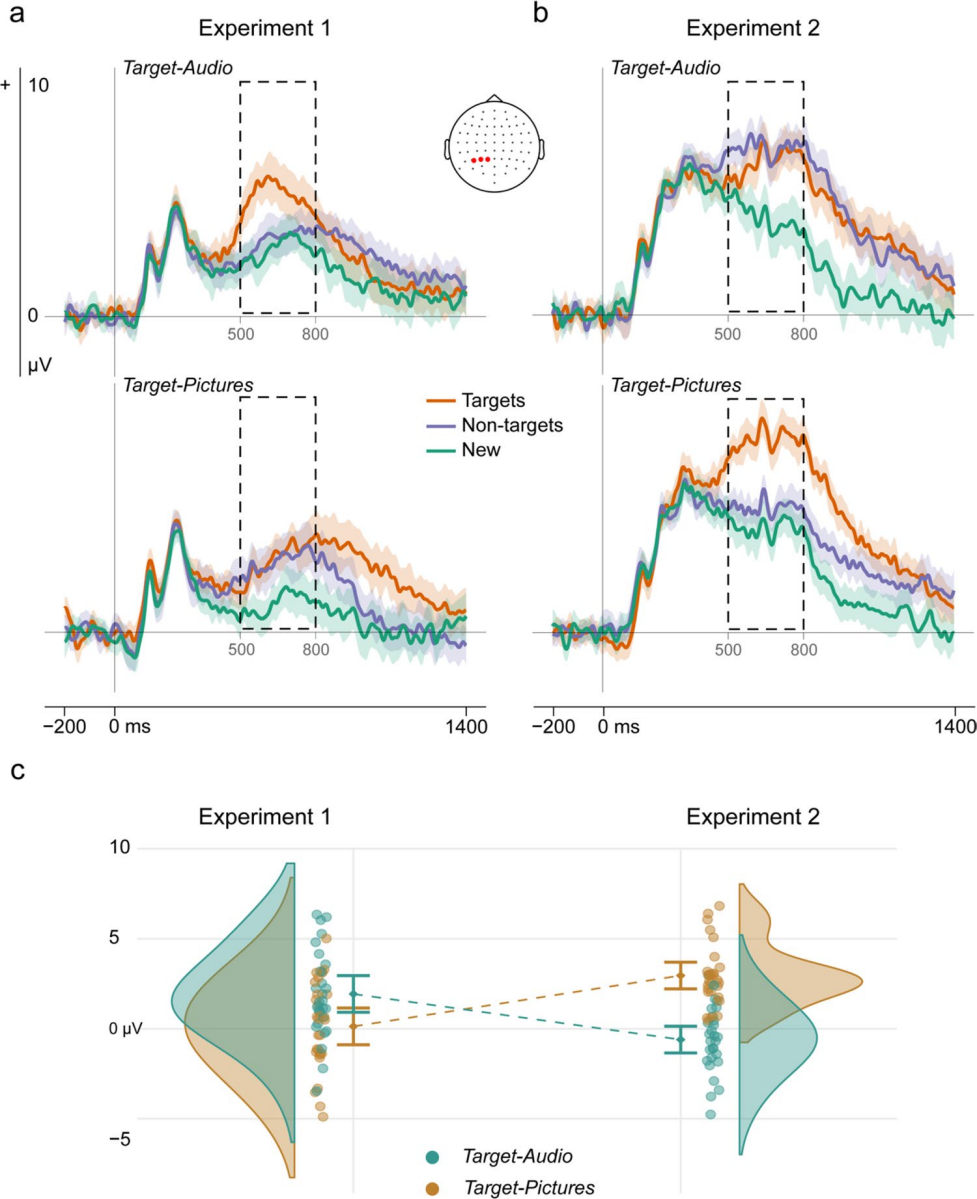
Selectivity of left parietal old/new effects. Mean grand-average ERP waveforms for target hits, non-target CRs, and new CRs over electrode sites (P1, P3, P5), plotted separately by Target Designation: (**a**) Experiment 1 with word cues; and (**b**) Experiment 2 with pictorial line drawing cues. The dashed areas indicate the analyzed time-window. The shaded areas represent the 95% confidence intervals for each time-point and adjusted using the Cousineau-Morey method for within-subject variables (Craddock, 2016). (**c**) Interaction effect of Target Designation x Cue Type (experiment) on the difference between left parietal ERPs evoked by target hits and non-target CRs. The colored dots are the difference scores for each participant, the shaded areas are the probability density function of the data, and error bars are the adjusted within-subject 95% confidence intervals around the means. Mean number of trials (range) contributing to ERPs in Experiment 1 for targets, non-targets, and new were 31 (14-37), 33 (18-39), and 35 (21-40) in the Target-Pictures block and 29 (18-38), 34 (25-40), and 33 (21-40) in the Target-Audio block. In Experiment 2, these were 33 (25-38), 32 (22-36), and 35 (25-40) in the Target-Pictures block and 25 (15-34), 34 (30-38), 29 (16-38) in the Target-Audio block

When retrieval cues were words (Experiment 1), left parietal old/new effects for targets were larger than those for non-targets only in the high cue-target overlap condition, when targets were auditory words, and not in the low cue-target overlap condition, when targets were pictures (Figures 2a, c). ANOVA with factors of Item Type (target hits/non-target CRs/new CRs) and Target Designation (picture/audio) showed significant main effects of Item Type *F*(1.91, 51.60) = 24.64, *p* < 0.001, *η*^*2*^_*p*_ = 0.477, and Target Designation *F*(1, 27) = 20.10, *p* = 0.001, *η*^*2*^_*p*_ = 0.427, as well as a significant interaction, *F*(1.83, 49.40) = 3.32, *p* = 0.048, *η*^*2*^_*p*_ = 0.110. Post hoc *t-*tests for the Target-Audio block showed that left parietal ERPs for target hits were significantly more positive than for both non-target, *t*(27) = 4.19, *p* < 0.001, *d* = 0.52 and new CRs, *t*(27) = 4.57, *p* < 0.001, *d* = 0.65, and the latter were statistically indistinguishable, *t*(27) = 0.95, *p* = 0.420, *d* = 0.13. In sharp contrast, in the Target-Pictures block left parietal ERPs evoked by both target hits and non-target CRs were significantly more positive-going than those for new CRs, *t*(27) = 4.77, *p* < 0.001, *d* = 0.64, and *t*(27) = 3.36, *p* = 0.004, *d* = 0.51, whereas target and non-target ERPs did not differ significantly, *t*(27) = 0.29, *p* = 0.775, *d* = 0.04. Thus, recollection as measured by the left parietal effect was selective for targeted information when participants were asked to endorse the studied auditory words but not selective when asked to endorse studied pictures.

We interpreted the results of Experiment 1 in terms of the higher overlap between word cues and the auditory source and, therefore, predicted a complementary asymmetry when retrieval cues were line drawings. The results of Experiment 2 supported this prediction (Fig. 2b, c). ANOVA with the same factors revealed a significant main effect of Item Type, *F*(1.55, 41.97) = 51.93, *p* < 0.001, *η*^*2*^_*p*_ = 0.658, a nonsignificant main effect of Target Designation *F*(1, 27) = 0.03, *p* = 0.874, *η*^*2*^_*p*_ = 0.001, and once again a significant interaction *F*(1.90, 51.21) = 20.98, *p* < 0.001, *η*^*2*^_*p*_ = 0.437. Post hoc *t*-tests showed that as expected, for the Target-Audio block ERPs to both target hits and nontarget CRs were significantly more positive than new CRs, *t*(27) = 5.16, *p* < 0.001, *d* = 0.57, and *t*(27) = 5.60, *p* < 0.001, *d* = 0.67. Target ERPs were nonsignificantly more *negative*-going than nontarget ERPs, *t*(27) = −1.74, *p* = 0.094, *d* = 0.13. In contrast, target prioritization was significant, and more pronounced, in the Target-Pictures block. Here, ERPs evoked by target hits were significantly larger than those for nontarget CRs, *t*(27) = 9.22, *p* < 0.001, *d* = 0.64, although both were significantly more positive than new CRs, *t*(27) = 9.25, *p* < 0.001, *d* = 0.84 for targets versus new, and *t*(27) = 2.55, *p* = 0.018, *d* = 0.21 for nontargets versus new. Thus, target recollection was not selective when auditory words were targets but prioritized when pictures were targets.

These apparent differences from Experiment 1 were confirmed in a direct comparison (Figure 2c). ANOVA with the additional between-participants factor of Cue Type (word cues/picture cues) on the difference between target and non-target ERPs revealed a significant interaction of Cue Type and Target Designation *F*(1, 54) = 38.04, *p* < 0.001, *η*^*2*^_*p*_ = 0.413. Post hoc *t-*tests confirmed that target and non-target left parietal effects differed more in the Target-Audio condition when cues were words (Experiment 1) as opposed to line drawings (Experiment 2), *t*(54) = 4.39, *p* < 0.001, *d* = 1.17, while selection in the Target-Picture condition was stronger when cues were line drawings, *t*(54) = −5.01, *p* < 0.001, *d* = 1.34.

#### Global analysis

**Fig. 3:**
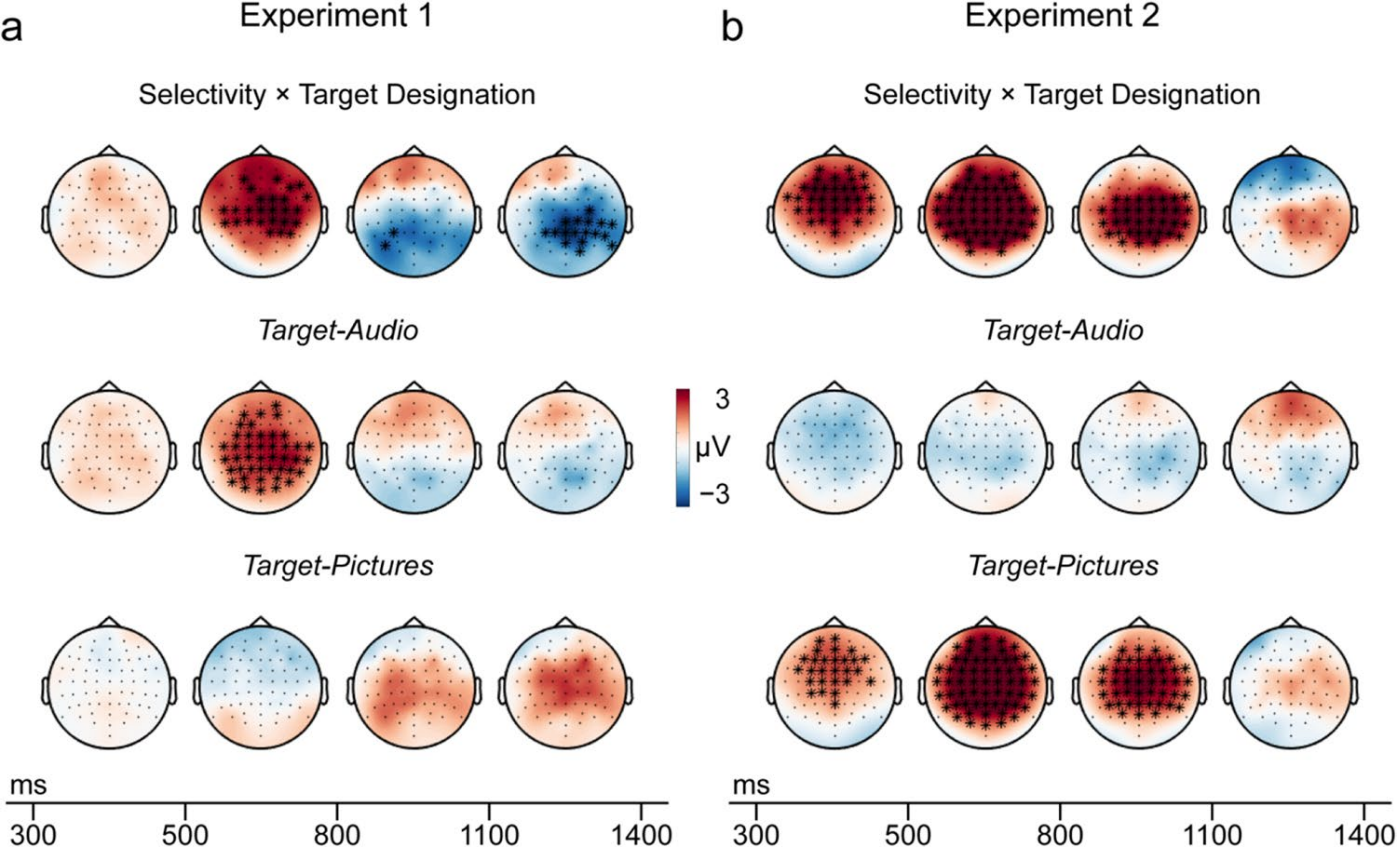
Global analyses of recollection selectivity: Target minus non-target ERPs. Topographic maps with significant clusters are shown for Experiment 1 (**a**) and Experiment 2 (**b**). Maps show ERP amplitude differences between target hits and non-target CRs by target designation (interaction at the top, Target-Audio in the middle, Target-Pictures at the bottom). Cluster significance is depicted by time-window, i.e., electrodes belonging to a significant cluster are highlighted (*) if significant on average in the plotted time-window.

The results converged with the focal analyses to reveal complementary differences in target versus non-target old/new effects according to target designation in both experiments, as well as a further, later-onsetting difference in Experiment 1 (Fig. 3). When visual words were cues (Experiment 1), the difference between target and non-target ERPs was significantly greater when participants targeted auditory words than pictures (*p* = 0.004). This interaction cluster was present between 451-874 ms and was widespread across the scalp. Therefore, although cluster tests do not provide precise spatial or temporal localization (Sassenhagen & Draschkow, 2019), the effect overlapped that shown in our focal analyses. Post hoc tests within blocks revealed significantly larger target than non-target ERPs (*p* = .002) when participants targeted auditory words. There was also an unexpected, later-onseting, significant interaction in the opposite direction in a cluster that was maximal over posterior electrodes and significant from 900-1,400 ms (*p* = .008). Post hoc tests revealed more positive-going target than non-target ERPs when participants targeted pictures (*p* = .020), but a non-significant reversed difference when participants targeted auditory words (*p* = .207).

In Experiment 2, as in the focal analysis, the difference between target and non-target ERPs was greater when pictures than auditory words were targets (*p* < 0.001). This effect was widespread over the scalp from 300-1,096 ms. Post hoc tests revealed that target and non-target ERPs did not differ reliably when participants used line drawings to target auditory words (*p* = 0.203); however, when the same cues were used to target pictures, ERPs were more positive-going for targets (*p* < .001; Fig. 3b). This interaction cluster therefore overlapped the effect shown in the focal analysis and took the same form.

### Retrieval goal states

#### Focal analysis

**Fig. 4:**
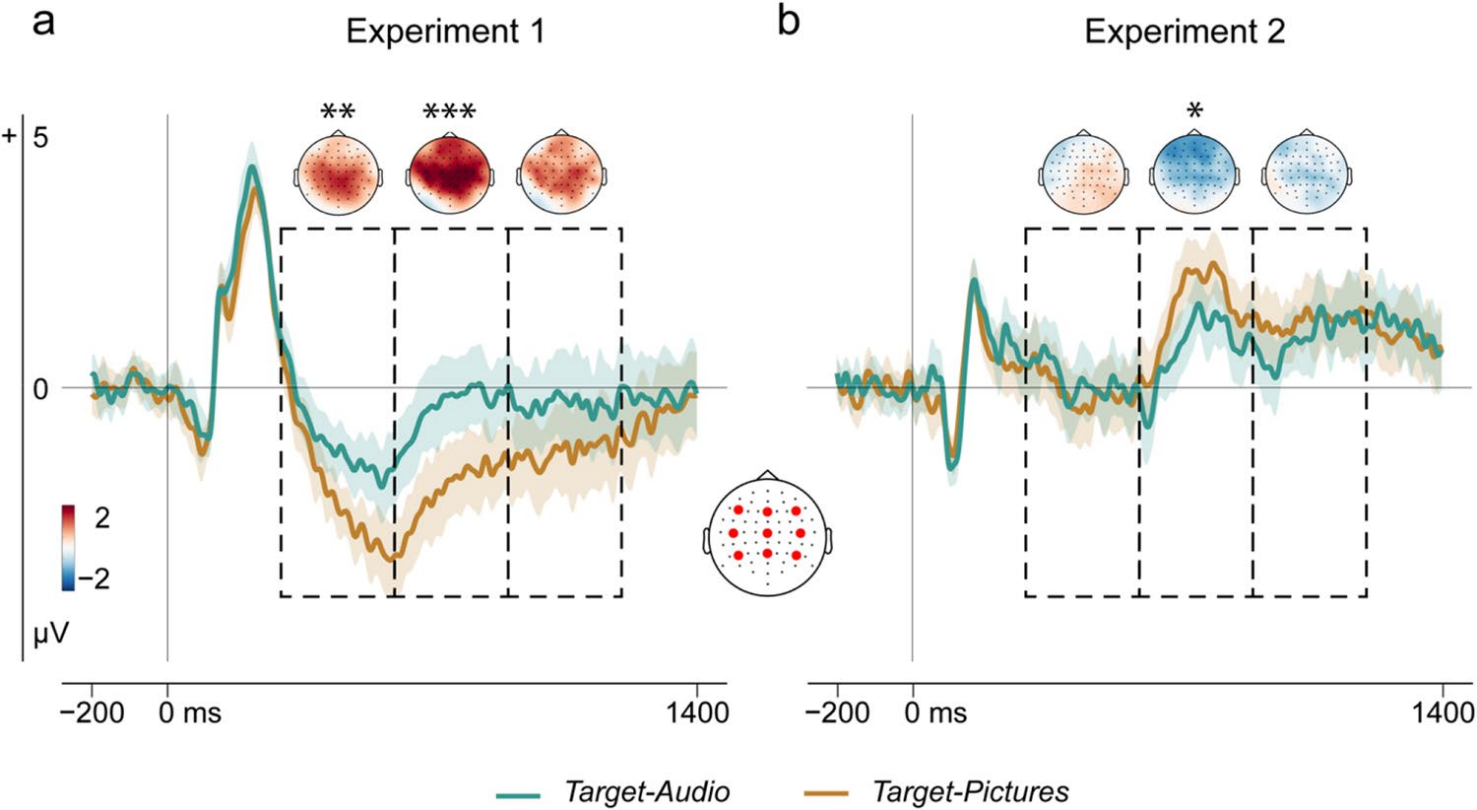
Retrieval goal states. Results of the focal analysis. Grand-average ERP waveforms for retrieval orientation effects are plotted for (**a**) Experiment 1, word cues, and (**b**) Experiment 2, pictorial cues. Data are averaged over the grid of 9 electrodes, highlighted in red. The dashed areas indicate the time-windows from 300-600, 600-900, and 900-1,200 ms, and time-windows with significant differences highlighted (*p ≤ .05, **p ≤ .01, ***p ≤ .001). The shaded areas show 95% confidence intervals for each time-point and adjusted using the Cousineau-Morey method for within-subject variables (Craddock, 2016). The upper parts of each panel show topographic maps of ERP amplitude differences between new CRs according to target designation (Target-Audio minus Target-Pictures) in each experiment. ^+^*p* ≤ .10; *p ≤ .05; **p ≤ .01; ***p ≤ 0.001.

Retrieval orientation ERP effects suggesting differences in retrieval goal states were present in both experiments and followed the predicted pattern, although they were smaller and had later onset in Experiment 2 (Fig. 4). When visual words were cues (Experiment 1), ERPs to new CRs were more positive-going in the Target-Audio than the Target-Picture condition from approximately 400-1,300 ms with a centroparietal scalp maximum. When pictures were cues (Experiment 2), these ERPs were more positive-going in the Target-Picture than the Target-Audio condition from approximately 600-1,100 ms, with a frontal scalp maximum. Thus, ERPs to unstudied items were more positive-going in the high cue overlap condition in each experiment.

In Experiment 1, ANOVA with factors of Target Designation (picture/audio), Hemisphere (left/midline/right), and Site (anterior/central/posterior) showed a significant main effect of Target Designation in Experiment 1 between 300-600 ms, *F*(1, 27) = 10.25, *p* = .003, *η*^*2*^_*p*_ = 275. This persisted from 600-900 ms, *F*(1, 27) = 12.76, *p* = .001, *η*^*2*^_*p*_ = 0.321, but was no longer reliable by 900-1,200 ms post-stimulus, *F*(1, 27) = 3.06, *p* = .091, *η*^*2*^_*p*_ = 0.102. In contrast, when cues were pictures (Experiment 2), retrieval orientation effects were significant only in the 600-900-ms time-window; for main effect of target designation, *F*(1, 27) = 4.39, *p* = .046, *η*^*2*^_*p*_ = 0.140; for 300-600 ms, *F*(1, 27) = 0.26, *p* = .613, *η*^*2*^_*p*_ = 0.01; for 900-1200 ms, *F*(1, 27) = 0.69, *p* = .413, *η*^*2*^_*p*_ = 0.025. Full ANOVA outputs are given in Supplemental Results available online (Table S1).

#### Global analysis

The results of the global retrieval orientation analyses also converged with those of the focal analyses. In Experiment 1, ERPs evoked by new CRs were more positive in the Target-Audio than the Target-Pictures block in a centroparietal cluster encompassing 403-920 ms (*p* = 0.002). However, for Experiment 2, the smaller retrieval orientation effects found in our focal analyses were not statistically significant in the global analysis (*p* = .064).

### Relations between retrieval goal states and recollection selectivity

**Fig. 5:**
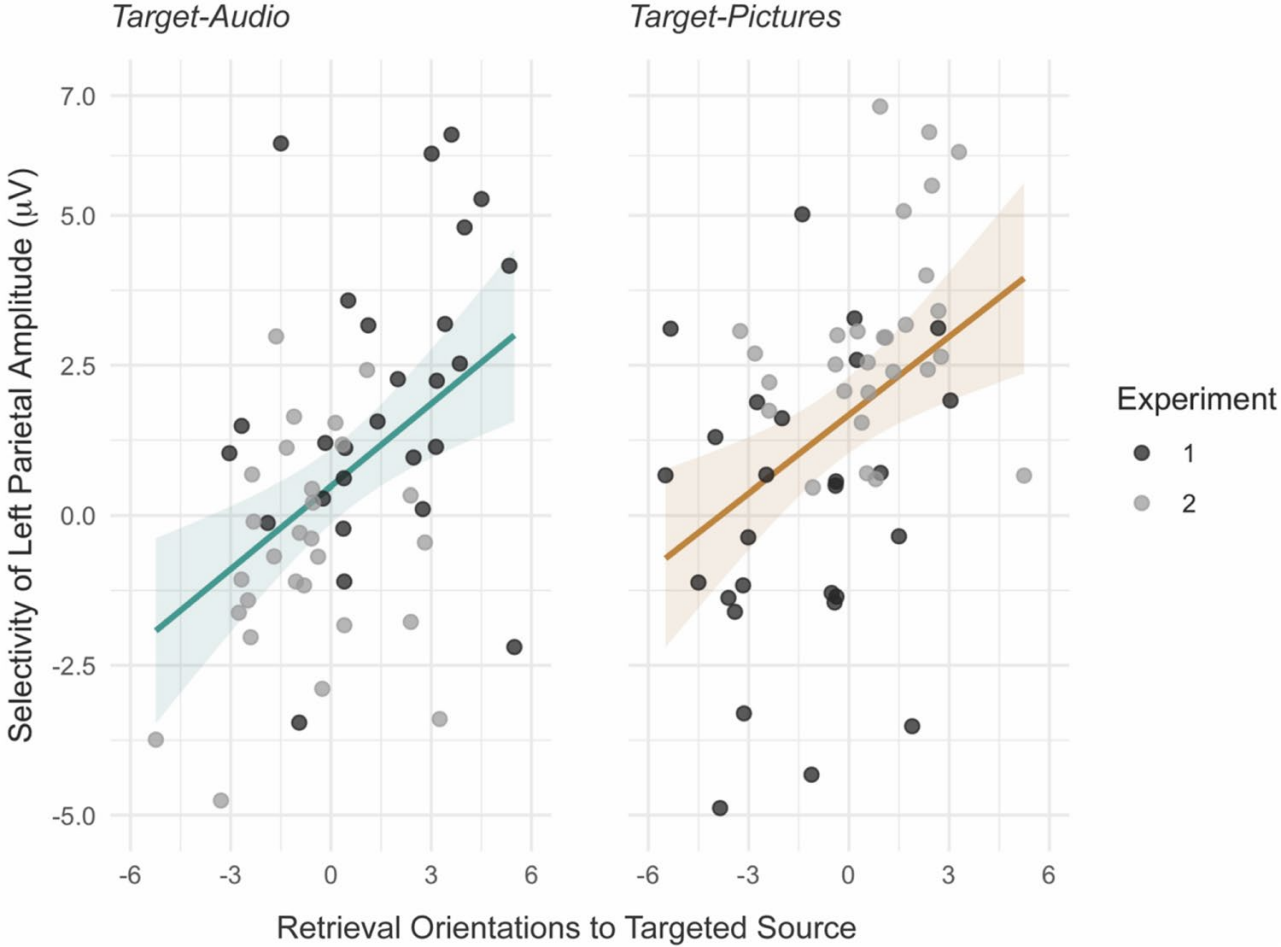
Relations between retrieval goal states and recollection selectivity. Panels show scatter plots of left parietal effect selectivity (*x*-axis: difference between target and non-target ERPs from 500-800 ms) against ERP retrieval orientation effects to the targeted source (*y*-axis: differences in new CR ERPs from 600-900 ms). Left panel: selectivity of the left parietal effect in the Target-Audio block, and Target-Audio minus Target-Pictures new CR ERPs. Right panel: selectivity of the left parietal effect in the Target-Pictures block, and Target-Pictures minus Target-Audio new CR ERPs. Data are pooled across Experiment 1 and Experiment 2. The lines represent the line of best fit and the shaded areas are the 95% confidence intervals

In exploratory analyses, we examined the relation between the retrieval goal states and recollection selectivity, as indexed by the left parietal ERP. If selective recollection is supported by the goal-related processes, we expected these two ERP measures to be positively correlated over participants, pooled over experiments (*N* = 56, FDR correction over 5 tests). First, we asked whether the degree to which participants oriented to each of the two sources correlated with their achieved recollection selectivity for that source. We obtained a measure of participants’ ability to orient toward the auditory source by subtracting the ERPs elicited by new CRs in the Target-Pictures block from those in the Target-Audio block between 600 to 900 ms (where the retrieval orientation effects were significant in both experiments). To examine orientation toward the picture source, we reversed the sign of this index. For each target designation, we then correlated the retrieval orientation effect with the difference in left parietal ERP magnitude between targets and non-targets. This revealed positive correlations between retrieval orientation effects and left parietal effect selectivity in both the Target-Audio block, *r* = .45, *t*(54) = 3.66, *p* = .003, and the Target-Picture block, *r* = .38, *t*(54) = 2.97, *p* = .011 (Fig. 5). These correlations did not differ between experiments and, therefore, did not depend significantly on the nature of the external cues (for Target-Audio, *r* = .29 for Experiment 1 and *r* = .20 for Experiment 2, *z* = 0.34, *p* = .735; for Target-Picture, *r* = .07 for Experiment 1 and *r* = .28 for Experiment 2, *z* = 0.77, *p* = .442). Thus, participants who exhibited a stronger retrieval orientation in favor of auditory information (more positive ERPs for new CRs in the Target-Audio than Target-Picture block) also showed a more selective left parietal ERP for auditory targets. Conversely, participants who adopted a stronger retrieval orientation in favor of pictorial information also showed more a selective left parietal effect for picture targets.

In a final set of exploratory correlational analyses, we explored the relationship between the ERP measures of recollection selectivity, retrieval orientation and behavior. To do this, we correlated the left parietal effect selectivity, and the magnitude of the auditory and picture retrieval orientation effects, with participants’ ability to discriminate between targets and non-targets (Source discrimination *d’*). We found no significant relationships (for left parietal effects: *r* = 0.14, *t*(54) = 1.00, *p* = .533; and for retrieval orientation effects in the Target-Audio block: *r* = 0.07, *t*(54) = 0.53, *p* = .748, and Target-Picture block, *r* = 0.04, *t*(54) = 0.30, *p* = 0.762).

## Discussion

Recollecting the past involves selecting from a large number of stored memory traces. We used ERPs to investigate how and when people recover desired information. By measuring time-resolved neural responses during recollection, we were able to quantify preretrieval selection more directly than it is possible with behavioral measures. In two pre-registered experiments, we manipulated the representational overlap between external retrieval cues and the information to be remembered. In Experiment 1, visual word test cues shared more processing with studied auditory words than studied pictures. The left parietal effect, an ERP marker of recollection, was selective — larger for targets than non-targets — only when auditory words were targets, and not when pictures were targets. Importantly, this asymmetric pattern of selectivity could not be explained by easier recollection of targets, as regardless of target designation, responses were slightly more accurate and faster to items studied as pictures. The data thus favored a cue-overlap account, suggesting that at least with word cues, pre-retrieval selection was effective only for the high-overlap (auditory word) source. In the second experiment, we directly tested this interpretation by changing the cues at test to picture line drawings, which would overlap more with the picture than the auditory source. As expected, we found target-selective left parietal effects only for the high-overlap (in this case picture) source.

These ERP asymmetries go beyond the findings of previous studies showing selectivity of the left parietal effect when targets were identical to the cues shown at test (Herron and Rugg 2003a; Stenberg et al., 2006). We show for the first time that the degree of cue-target overlap has downstream consequences for successful retrieval even when overlap is incomplete, as we found selective left parietal effects when participants’ memory was probed with test cues that were not identical “copy cues” of studied items, and for both verbal and pictorial cues (see also Czernochowski et al., 2005). Together, the data support the view that cue-target overlap is a critical factor enabling selection prior to recollection. These findings extend support for the longstanding principle of encoding specificity, which assumes that cues trigger recollection when they elicit representations that overlap with stored memory traces (Tulving & Thomson, 1973; see also Morris, Bransford, & Franks, 1977). This principle is qualified by evidence that it is “diagnostic” rather than absolute overlap that must be maximized, meaning that effective cues are those that overlap more with the targeted *relative* to the non-targeted information (Goh & Lu, 2012; Nairne, 2002). In the current experiments, we increased diagnosticity and cue-target overlap in tandem by reducing cue-non-target overlap at the same time. A limitation is therefore that we were unable to test this further proposal. This will be an important goal for future studies.

Given a certain external cue, how is recollection of targets prioritized? Theoretical models of recollection assume that selection is achieved by processing the available cue according to our goals (Anderson & Bjork, 1994; Burgess & Shallice, 1996; Rugg & Wilding, 2000). In both the current experiments, differences in left parietal effect selectivity were accompanied by ERP differences reflecting retrieval orientation on new CR trials where no retrieval took place. These goal-related ERPs were more positive-going in the conditions with the greatest cue-target overlap, extending previous findings from the studies with one copy-cue condition (Herron & Rugg, 2003a; Hornberger et al., 2004; Morcom & Rugg, 2012; see also Bramão et al., 2017). As noted earlier, Dzulkifli et al. (2006) and Dzulkifli and Wilding (2005) previously demonstrated retrieval orientation ERP effects only when recollection was selective and abolished both of these goal-related effects and selective recollection by increasing target retrieval difficulty (but see Rosburg et al., 2013, 2014). However, our data confirm that the difficulty account is insufficient, because selectivity did not track the ease of target retrieval in the current study. The cue overlap account could explain Dzulkifli and Wilding (2005) and Dzulkifli et al. (2006) findings if the “difficulty” manipulations prevented proactive goal-directed processes from generating effective cues for one source: for example, because longer study lists make source cues less diagnostic. This proposal remains to be tested.

Further evidence that selective recollection is achieved via goal-directed control processes comes from our finding that retrieval orientation effects correlated positively with the degree of selectivity. Across experiments, participants who exhibited larger retrieval orientation effects for the targeted source also showed more target-selective left parietal effects. Thus, larger positive-going ERPs for the currently targeted source were associated with more selective recollection of that source (as indexed by the left parietal ERP), regardless of whether external cues had high or low target overlap. The same goal-related positivity also was present, on average, in the high relative to the low cue overlap condition in each experiment (Fig. 4), consistent with our interpretation that these average ERP retrieval orientation effects reflected additional processing in the high overlap conditions. Interestingly, we found no significant relationships between source discrimination performance in the exclusion task and the ERP measures of retrieval orientation and recollection selectivity. Although positive associations might be expected, relations between individual retrieval processes and performance are likely to be complex. A previous study did not detect significant associations between the left parietal effect and simple recognition or subjective recollection measures in substantial samples (MacLeod & Donaldson, 2017). We and others have found positive associations between target-selectivity of the left parietal ERP and measures of working memory capacity, supporting the proposal that selectivity reflects memory control ability (Elward et al., 2013; Elward & Wilding, 2010; Keating et al., 2017). Because mnemonic decisions occur at the end of the retrieval process, a more complex model that accounts for the contribution of pre-retrieval control, working memory, and cue overlap is likely to be needed to understand the relationship between the left parietal effect and mnemonic performance. This will be an important goal for future studies.

Another reason why close coupling of target-selectivity and performance may not be observed is the availability of alternative retrieval strategies. This is a limitation of the current study and of most other imaging studies of recollection selectivity using the exclusion task. Here, some participants exhibited “negative” retrieval orientation effects (Figure 5), suggesting possible orientation to the non-targeted source information. This may indicate that not all participants adopted a target-selective strategy. Instead, some may have attempted to prioritize non-target recall in at least one condition — a “recall-to-reject” strategy that would be expected to elicit a non-selective or even non-target-selective left parietal effect (Rosburg & Mecklinger, 2017). Although this strategy diverges from the task instructions, it can support adequate discrimination performance in this typical exclusion task with only two alternative sources. However, although some use of recall-to-reject can explain partial selectivity at the group level, it cannot explain away our main finding of complementary patterns of selectivity depending on the overlap between external cues and targeted information. Future studies could constrain the available strategies by increasing the number of targeted sources — a manipulation that has been shown to prevent effective recall-to-reject (Gallo, 2004).

A number of questions remain about the nature of the goal-driven processing engaged in response to the external memory cues. The encoding specificity principle predicts that people will seek to maximize diagnostic overlap by mentally reinstating representations stored in targeted but not non-targeted memories. This increase in cue overlap could be achieved by at least two means: elaborating or constraining cue processing (Hornberger et al., 2004). For example, in Experiment 1, participants may have elaborated on the visual word cues by emphasizing the phonological features shared with auditory studied items, increasing cue-target overlap. Alternatively, or in addition, they may have constrained cue processing to *decrease* overlap with non-targeted memory representations, for example by suppressing imagery processes that would overlap with non-targeted picture representations in Experiment 1. The present data cannot test these two non-exclusive mechanisms but support for some form of mental reinstatement comes from multivariate imaging studies. McDuff and others (2009) used fMRI with an exclusion task similar to those used in the ERP studies by Dzulkifli et al. (2005; 2006) and Evans et al. (2010). At test, when participants oriented to any one of three sources defined by different study phase orienting tasks, distributed multivoxel activity patterns over the whole brain resembled the patterns elicited by the encoding operations associated with the targeted source (McDuff et al., 2009). Neural reinstatement of representations of study phase temporal context (Kragel et al., 2021; Manning et al., 2011) or semantic content (Kragel et al., 2021) also have been demonstrated using intracranial EEG during the final second of a memory search period, just before free recall, and shown to predict the dynamics of recall performance. While it is difficult to definitively separate memory search from initial successful retrieval in free recall, the findings provide initial support for the assumption that mental reinstatement occurs before retrieval (see also Polyn, 2005). However, to our knowledge, no study has yet linked this mental reinstatement to selection of which items will be recollected.

Although our data demonstrate clearly complementary patterns of selective recollection of the two sources under different retrieval cues, questions also remain about the degree of selection achieved. While in Experiment 1, recollection as measured using the left parietal ERP effect appeared to be completely selective for auditory word targets, in Experiment 2 the same marker suggested incomplete selectivity in the corresponding high-overlap picture target condition (Fig. 2). There is no reason to think that selective remembering is an all-or-none phenomenon. Although prior ERP studies that used visual word copy-cues to target studied visual words (Herron & Rugg, 2003a; Stenberg et al., 2006) did not detect a non-target left parietal ERP effect when non-targets were pictures, an fMRI study using the same task revealed a more nuanced picture. Although only targets elicited old/new effects in left angular gyrus (a possible source of the left parietal ERP effect), non-target old/new effects were detected in other brain regions (Morcom & Rugg, 2012). The findings of Experiment 1 echo this earlier result but add additional temporal information: although the left parietal effect was selective in the target-audio condition with no detectable effect for non-targets, the global analysis showed that there was some processing of non-targets after 800-ms post-stimulus. This later-onsetting activity presumably reflected post-retrieval processing. Other studies have reported incomplete selectivity of the left parietal effect (Rosburg & Mecklinger, 2017). Further research is needed to determine whether some elements of memories are more easily selected than others and to understand potential trade-offs between proactive pre-retrieval processing and reactive post-retrieval processing that may have consequences for populations who are less able to remember selectively (Morcom, 2016).

The foregoing arguments assume that the left parietal ERP effect can be used to index recollection success. While relations between the left parietal effect and both objective and subjective recollection indices are well established (see *Introduction*), some researchers have questioned how directly this ERP reflects mnemonic processes. Yang et al. (2019) found a significant left parietal effect for previously presented word items in a recent-exposure recognition task but not in a lifetime frequency judgment task and suggested that this ERP might reflect decision-making rather than mnemonic processes (O’Connor et al., 2010 for a similar interpretation of left parietal activation using fMRI). However, the latter finding can be explained if people did not recollect the recently presented words in the lifetime frequency task, consistent with the established association between the left parietal ERP effect and recollection as opposed to familiarity-based memory (Friedman & Johnson, 2000; Rugg & Curran, 2007). The current pattern of left parietal selectivity findings also cannot be explained solely in terms of decision-related processes, as the presence of the left parietal effect for non-targeted items tracked the relation between targets (and non-targets) and retrieval cues. Similarly, measures of response bias in our experiments tracked the overall memory advantage for the picture source, so decision processes associated with criterion shifts cannot explain the pattern of selectivity.

Another concern has been that the left parietal ERP is sensitive to the strength of the memory signal, rather than indexing a pure recollection process (Brezis et al., 2017, but see Horne et al., 2020). However, regardless of whether recognition reflects one or two underlying processes (Dunn, 2004; Rugg & Curran, 2007), the current modulations of the left parietal effect show that pre-retrieval selection has downstream consequences on retrieval success. Given that recovery of source information is involved in discriminating between target and non-targets in this task, we interpret our data in terms of recollection selectivity. We do this with the caveat that our task did not directly measure recollection, so interpreting the left parietal ERP as a recollection signal involves “reverse inference.” Although this reasoning is frequently ill-founded (Poldrack, 2006), it is less problematic when done in a task context similar to those in which the original association between the neural signal and the cognitive process was established (Klein, 2012) – in this case, a recognition memory task. A more specific concern is that the smaller left parietal effect for non-targets than for targets might be a consequence of forgetting, rather than pre-retrieval selection, because if an item is experienced as unstudied it will be judged to be a “non-target.” However, if this were the sole explanation for target-non-target left parietal ERP differences, we would expect to see some reduction in the size of the left parietal effect for non-targets relative to targets but should still find reliable differences relative to new items, for which memory is absent — unlike in Experiment 1 (Wilding & Rugg, 1997; Rosburg & Mecklinger, 2017). Moreover, while forgetting may explain some instances where the ease of target recollection tracks selectivity of the left parietal ERP, it cannot explain the present findings in relation to external retrieval cues. Here, selectivity was not only present when non-target memory was better than target memory, but also tracked the degree of cue-target overlap even when (in Experiment 1) overlap was higher in the condition in which target recollection was lower.

## Conclusions

These experiments are the first to show that recollection is selective when retrieval cues overlap more closely with sought-for information in memory, implicating pre-retrieval control. When recollection is selective, neural activity associated with retrieval goal states is more pronounced. The data open up several new possibilities for future research into the goal-states that enable selective remembering, its implementation, and consequences for mnemonic experience.

## Data Availability

The stimuli, task materials and data are publicly available and can be accessed online.

## Supplemental Information

ESM 1 Supplemental materials and results are available online and can be accessed at https://osf.io/8pt25. (PDF 266 kb)

## Code availability

Task and analysis scripts are publicly available and can be accessed online.
