# Correction to: Cue overlap supports preretrieval selection in episodic memory: ERP evidence

**DOI:** 10.3758/s13415-022-00995-0

**Published:** 2022-03-15

**Authors:** Arianna Moccia, Alexa M. Morcom

**Affiliations:** School of Psychology, University of Sussex, Pevensey I, Falmer, BN1 9QH UK

**Correction to: Cogn Affect Behav Neurosci**

10.3758/s13415-021-00971-0

This article was updated to correct the digits reported for one of the exploratory correlational analyses.

