# Supplementary material for: Cue overlap supports preretrieval selection in episodic memory: ERP evidence
Source: Cogn Affect Behav Neurosci. 2021 Dec 29;22(3):492–508. doi: 10.3758/s13415-021-00971-0 (PMC9090896; doi:10.3758/s13415-021-00971-0)
Supplement: ESM 1 — Supplemental materials and results are available online and can be accessed at https://osf.io/8pt25. (PDF 266 kb) [file 13415_2021_971_MOESM1_ESM.pdf]

Supplemental Materials

Supplemental image processing

Line drawings were created by first applying a 5 Hz high-pass filter to the images in Photoshop. Filtered images were then transformed to black and white drawings, and the white background was set as the alpha channel so that the images appeared as black line drawings on a transparent background. Image processing on filtered pictures was achieved in Matlab 2018a. In Experiment 1, 76 pictures were taken from the BOSS database (Brodeur et al., 2014), 68 from the POPORO database (Kovalenko et al., 2012), and 96 were sourced online. In Experiment 2, seven practice items were swapped to the main experimental task to improve image quality of test cues. Of these, four additional images were taken the POPORO database (Kovalenko et al., 2012) and three were sourced online.

Supplemental EEG pre-processing

Thresholds for automated artifact rejection

The pre-registered EEG preprocessing pipeline and artefact rejection criteria can be found at osf.io/j84z6 and osf.io/pqn4z (see EEG recording and pre-processing for more details). We used customized functions from the FASTER toolbox (Nolan et al., 2010) to identify and reject epochs with excessive motion artefacts, drifts, or gross EOG movements, and channels with excessive noise. For each participant, we rejected epochs whose *z*-transformed values exceeded $\pm$ 3 over trials. These values were each epoch’s amplitude range, variance, and deviation from each channel’s mean value. The latter measure was computed by subtracting each channel’s mean amplitude within the epoch from the channel’s mean amplitude across epochs, which was then averaged across all channels. Channels’ thresholds were computed by calculating

each channel's mean correlation with all other channels, variance, and Hurst exponent, a measure of a signal's long-range dependence.

Supplemental Results

Discrimination performance in the Recognition Exclusion Task

In a separate preregistered analysis of memory performance using discrimination (*d'*) measures to correct for response bias, we calculated *d'* and criterion (C) for item memory and non-target false recognition for the Target-Picture and Target-Audio blocks (Snodgrass & Corwin, 1988). Item *d'* was calculated by subtracting the z-scored proportion of new FAs from the *z*-scored proportion of target hits. Similarly, nontarget *d'*, a measure of non-target false recognition, was obtained by subtracting the *z*-new FAs score to *z*-nontarget FAs score. These were corrected for a potential outcome of zero as in Hautus (1995) and Snodgrass & Corwin (1988) and response bias (C) calculated as in Macmillan and Creelman (1991) (see main manuscript for details). Confidence intervals (CIs) reported below are adjusted using the Cousineau-Morey method for within-subject variables (Morey, 2008).

The results of the *d*' analysis converged with those of the analysis of response proportions reported in the main paper (see Exclusion Task Performance). We examined whether participants' mnemonic discrimination scores and their response bias differed according to target designation with paired-sample *t*-tests. Memory was consistently better for pictures compared to auditory words. In Experiment 1, participants' item memory was better for pictures ($M = 2.40$, 95% CI = 0.23) than auditory words ($M = 2.05$, 95% CI = 0.23), $t(27) = 2.16$, $p = .040$, Cohen's $d = 0.58$, whereas their response bias criterion was not different when participants recognized targets studied as auditory words ($M = 0.22$, 95% CI = 0.09) as opposed to pictures ($M = 0.26$, 95% CI = 0.09), $t(27) = 0.51$, $p = .613$, , Cohen's $d = 0.14$. There was also a significant increase

in false recognition of non-targets when these had been studied as auditory words (*M* = 0.26, 95% CI = 0.20) as opposed to pictures (*M* = - 0.14, 95% CI = 0.20), *t*(27) = 2.97, *p* = .006, Cohen's *d* = 0.79. Once again, participants' response bias criterion was not different for non-targets that were studied as auditory words (*M* = 1.33, 95% CI = 0.14) versus pictures (*M* = 1.32, 95% CI = 0.14), *t*(27) = 0.06, *p* = .955, , Cohen's *d* = 0.02.

In Experiment 2, there was again consistently better memory for pictures than auditory words. Here, item memory was significantly better when participants targeted pictures (item memory: *M* = 2.65, 95% CI = 0.20) versus auditory words (item memory: *M* = 1.58, 95% CI = 0.20), *t*(27) = 7.70, *p* < .001, Cohen's *d* = 2.06. As in Experiment 1, participants' response bias criteria did not significantly differ when participants identified targets studied as pictures (*M* = 0.08, 95% CI = 0.09) as opposed to auditory words (*M* = 0.20, 95% CI = 0.09), *t*(27) = 1.90, *p* = .068, Cohen's *d* = 0.51. This increased mnemonic discrimination accuracy for pictures than auditory words was consistent with the greater overlap of test cues with the picture source. Once again, false recognition was greater for items that that been studied as auditory words (*M* = 0.19, 95% CI = 0.20) as opposed to pictures (*M* = − 0.60, 95% CI = 0.25), *t*(27) = 5.67, *p* < .001, , Cohen's *d* = 1.51, but the participants' response bias criteria was not different when non-targets were studied as pictures (in the Target-Audio block, *M* = 1.29, 95% CI = 0.10) than as auditory words (in the Target-Picture block, *M* = 0.31, 95% CI = 0.10), *t*(27) = 0.23, *p* = .820, Cohen's *d* = 0.06.

Retrieval goal states supplemental results

Full ANOVA results of focal analysis.

To investigate retrieval orientation effects indexing retrieval goal states, we analyzed ERPs elicited by new CRs according to target designation in a focal analysis (see ERP results in main manuscript for details). Full ANOVA outputs are given in Table S1.

Table S1. Focal Retrieval Orientation Analyses: Omnibus Repeated Measures ANOVAs

		Experiment 1					Experiment 2				
Time window (ms)	Effect	df	MSE	*F*	η^2_p	*p*	df	MSE	*F*	η^2_p	*p*
**300 - 600**											
	Target Designation	1, 27	12.30	10.25 **	.275	.003	1, 27	19.38	0.26	.010	.613
	Hemisphere	1.93, 52.00	9.72	5.89 **	.179	.005	1.97, 53.19	13.83	1.66	.058	.200
	Site	1.11, 30.09	52.03	40.88 ***	.602	<.001	1.08, 29.12	75.20	90.66 ***	.771	<.001
	Target Designation×Hemisphere	1.97, 53.30	1.42	0.43	.016	.652	1.88, 50.75	1.28	3.18 +	.105	.053
	Target Designation×Site	1.22, 32.81	3.12	1.75	.061	.196	1.34, 36.17	1.93	1.63	.057	.212
	Hemisphere×Site	2.85, 76.83	1.86	31.11 ***	.535	<.001	3.16, 85.39	3.24	7.17 ***	.210	<.001
	Target Designation×Hemisphere×Site	3.47, 93.62	0.27	0.85	.030	.485	3.13, 84.59	0.58	0.27	.010	.858
**600 - 900**											
	Target Designation	1, 27	27.81	12.76 **	.321	.001	1, 27	17.16	4.39 *	.140	.046
	Hemisphere	1.99, 53.85	15.30	4.04 *	.130	.023	1.95, 52.68	13.77	0.87	.031	.425
	Site	1.21, 32.59	30.10	60.96 ***	.693	<.001	1.07, 28.90	62.18	18.07 ***	.401	<.001
	Target Designation×Hemisphere	1.84, 49.56	2.42	0.12	.004	.872	1.99, 53.66	1.59	0.33	.012	.717
	Target Designation×Site	1.27, 34.34	3.64	1.36	.048	.260	1.31, 35.49	3.57	1.13	.040	.312
	Hemisphere×Site	2.23, 60.09	4.11	29.32 ***	.521	<.001	3.12, 84.21	4.24	30.44 ***	.530	<.001
	Target Designation×Hemisphere×Site	3.62, 97.63	0.50	0.75	.027	.547	3.31, 89.37	0.66	0.58	.021	.646
**900 - 1,200**											
	Target Designation	1, 27	37.53	3.06 +	.102	.092	1, 27	21.04	0.69	.025	.413
	Hemisphere	1.95, 52.56	13.73	10.42 ***	.278	<.001	1.87, 50.57	14.30	0.95	.034	.388
	Site	1.25, 33.64	18.44	28.16 ***	.511	<.001	1.08, 29.16	41.41	4.85 *	.152	.033
	Target Designation×Hemisphere	1.94, 52.46	1.94	0.19	.007	.819	1.94, 52.47	2.33	0.06	.002	.943
	Target Designation×Site	1.65, 44.63	2.50	1.38	.049	.260	1.43, 38.58	3.93	0.07	.003	.874
	Hemisphere×Site	2.72, 73.54	3.42	18.83 ***	.411	<.001	3.46, 93.45	3.35	31.02 ***	.535	<.001
	Target Designation×Hemisphere×Site	3.57, 96.51	0.62	1.24	.044	.299	3.70, 99.97	0.79	1.32	.046	.271

+ $p \leq .10$, * $p \leq .05$, ** $p \leq .01$, *** $p \leq .001$.

Preparatory cue ERP analyses.

Further pre-registered analyses of retrieval orientation effects examined differential preparatory activity when participants were preparing to retrieve according to target designation (i.e, when pictures were targets versus when auditory words were targets, see Materials and Procedure in main manuscript). These ERPs were time-locked to pre-cues that were followed by a correct response to the subsequent trial in the Target-Picture and Target-Audio block. The mean number of trials and range that contributed to these ERPs were 98.46 (65-112) and 96.43 (69-113) for Target-Picture and Target-Audio pre-cues, respectively, in Experiment 1 and 102.31 (87-115) and 91.66 (75-106) in Experiment 2. In a focal analysis, we examined ERPs over a grid of 6 frontal electrodes (F1, F3, F5/F2, F4, F6) in four 250 ms time-windows from 0-1,000 ms, following Herron (2018). ANOVAs with factors of Target Designation (picture/audio) and Hemisphere (left/right) did not yield any significant results including the factor of Target Designation (for full results see Table S2). In the global analysis across all scalp electrodes and time-points in the preparatory time-window (0-2,300 ms), we used a cluster-based permutation *t*-test to correct for multiple comparisons (Maris & Oostenveld, 2007; Oostenveld et al., 2011, see Global ERP analyses in main manuscript). These analyses also did not show any significant effects of Target Designation (at 1-tailed cluster-level α of .025).

Table S2. Focal Repeated Measures ANOVAs: Preparatory Cue Effects

		Experiment 1					Experiment 2				
Time window (ms)	Effect	df	MSE	*F*	η^2_p	*p*	df	MSE	*F*	η^2_p	*p*
**0 - 250**											
	Target Designation	1, 27	1.04	0.97	.035	.333	1, 27	0.91	0.14	.005	.712
	Hemisphere	1, 27	0.14	8.22 **	.233	.008	1, 27	0.08	1.38	.049	.251
	Target Designation×Hemisphere	1, 27	0.05	0.01	<.001	.913	1, 27	0.06	0.97	.035	.335
**250 - 500**											
	Target Designation	1, 27	1.93	0.01	<.001	.907	1, 27	1.93	1.29	.046	.266
	Hemisphere	1, 27	0.22	19.85 ***	.424	<.001	1, 27	0.39	1.91	.066	.178
	Target Designation×Hemisphere	1, 27	0.16	0.23	.008	.636	1, 27	0.14	0.49	.018	.491
**500 - 750**											
	Target Designation	1, 27	1.86	0.12	.004	.737	1, 27	1.44	0.30	.011	.590
	Hemisphere	1, 27	0.29	25.51 ***	.486	<.001	1, 27	0.26	8.82 **	.246	.006
	Target Designation×Hemisphere	1, 27	0.15	0.00	<.001	.971	1, 27	0.18	1.08	.038	.308
**750 - 1,000**											
	Target Designation	1, 27	2.17	0.01	<.001	.907	1, 27	1.86	2.08	.071	.161
	Hemisphere	1, 27	0.39	17.88 ***	.398	<.001	1, 27	0.23	10.42 **	.278	.003
	Target Designation×Hemisphere	1, 27	0.14	0.04	.001	.851	1, 27	0.23	0.73	.026	.400

+ $p \leq .10$, * $p \leq .05$, ** $p \leq .01$, *** $p \leq .001$.

References

Brodeur, M. B., Guérard, K., & Bouras, M. (2014). Bank of Standardized Stimuli (BOSS) Phase II: 930 New Normative Photos. *PLoS ONE*, *9*(9), e106953. https://doi.org/10.1371/journal.pone.0106953

Kovalenko, L. Y., Chaumon, M., & Busch, N. A. (2012). A Pool of Pairs of Related Objects (POPORO) for Investigating Visual Semantic Integration: Behavioral and Electrophysiological Validation. *Brain Topography*, *25*(3), 272–284. https://doi.org/10.1007/s10548-011-0216-8

Hautus, M. J. (1995). Corrections for extreme proportions and their biasing effects on estimated values ofd′. *Behavior Research Methods, Instruments, & Computers*, *27*(1), 46–51. https://doi.org/10.3758/BF03203619

Herron, J. E. (2018). Direct electrophysiological evidence for the maintenance of retrieval orientations and the role of cognitive control. *NeuroImage*, *172*, 228–238. https://doi.org/10.1016/j.neuroimage.2018.01.062

Macmillan, N. A., & Creelman, C. D. (1991). Detection theory: A user's guide. Cambridge: Cambridge University Press.

Maris, E., & Oostenveld, R. (2007). Nonparametric statistical testing of EEG- and MEG-data. *Journal of Neuroscience Methods*, *164*(1), 177–190. https://doi.org/10.1016/j.jneumeth.2007.03.024

Morey, R. D. (2008). Confidence Intervals from Normalized Data: A correction to Cousineau (2005). *Tutorials in Quantitative Methods for Psychology*, *4*(2), 61–64. https://doi.org/10.20982/tqmp.04.2.p061

Nolan, H., Whelan, R., & Reilly, R. B. (2010). FASTER: Fully Automated Statistical Thresholding for EEG artifact Rejection. *Journal of Neuroscience Methods*, *192*(1), 152–162. https://doi.org/10.1016/j.jneumeth.2010.07.015

Oostenveld, R., Fries, P., Maris, E., & Schoffelen, J.-M. (2011). FieldTrip: Open Source Software for Advanced Analysis of MEG, EEG, and Invasive Electrophysiological Data. *Computational Intelligence and Neuroscience*, *2011*. https://doi.org/10.1155/2011/156869

Snodgrass, J. G., & Corwin, J. (1988). Pragmatics of Measuring Recognition Memory: Applications to Dementia and Amnesia. *Journal of Experimental Psychology: General*, *117*(1), 34–50.
